# iASPP regulates neurite development by interacting with Spectrin proteins

**DOI:** 10.3389/fnmol.2023.1154770

**Published:** 2023-05-22

**Authors:** Junhao Wang, Chunhong Jia, Qiong Gao, Jiwen Zhang, Xi Gu

**Affiliations:** ^1^Fujian Key Laboratory for Translational Research in Cancer and Neurodegenerative Diseases, Institute for Translational Medicine, School of Basic Medical Sciences, Fujian Medical University, Fuzhou, China; ^2^Department of Neonatology, The Third Affiliated Hospital of Guangzhou Medical University, Guangzhou, China

**Keywords:** iASPP, Sptan1, Sptbn1, phosphorylation modification, neurite development

## Abstract

**Introduction:**

Since its discovery in 1999, a substantial body of research has shown that iASPP is highly expressed in various kinds of tumors, interacts with p53, and promotes cancer cell survival by antagonizing the apoptotic activity of p53. However, its role in neurodevelopment is still unknown.

**Methods:**

We studied the role of iASPP in neuronal differentiation through different neuronal differentiation cellular models, combined with immunohistochemistry, RNA interference and gene overexpression, and studied the molecular mechanism involved in the regulation of neuronal development by iASPP through coimmunoprecipitation coupled with mass spectrometry (CoIP-MS) and coimmunoprecipitation (CoIP).

**Results:**

In this study, we found that the expression of iASPP gradually decreased during neuronal development. iASPP silencing promotes neuronal differentiation, while its overexpression inhibited neurite differentiation in a variety of neuronal differentiation cellular models. iASPP associated with the cytoskeleton-related protein Sptan1 and dephosphorylated the serine residues in the last spectrin repeat domain of Sptan1 by recruiting PP1. The non-phosphorylated and phosphomimetic mutant form of Sptbn1 inhibited and promoted neuronal cell development respectively.

**Conclusion:**

Overall, we demonstrate that iASPP suppressed neurite development by inhibiting phosphorylation of Sptbn1.

## Introduction

P53 is a well-studied tumor suppressor that prevents tumor development and progression by promoting apoptosis and cell cycle arrest. The activity of p53 is regulated by multiple mechanisms (Hafner et al., 2019). The apoptosis-stimulating protein of p53 (ASPP) family proteins are important proteins that regulate the proapoptotic function of p53 (Samuels-Lev et al., 2001). The ASPP protein family includes ASPP1, ASPP2 and inhibitor of ASPP (iASPP) (Sullivan and Lu, 2007). While ASPP1 and ASPP2 enhance the proapoptotic effect of p53 by directly binding to p53, the binding of iASPP with p53 antagonizes this role (Samuels-Lev et al., 2001). iASPP is upregulated in a variety of tumors and promotes tumor cell survival (Liu et al., 2008; Cai et al., 2012). While most studies of ASPP family proteins have focused on tumors, the role played by ASPP proteins in other pathologies and in normal tissues has gradually emerged. One study found that iASPP interacts with desmoplakin and desmin in cardiomyocytes and regulates desmosomal integrity and intermediate filament organization. Dysregulation of iASPP leads to arrhythmic right ventricular cardiomyopathy (Notari et al., 2015). iASPP is also involved in the development of skin, and iASPP and p63 are involved in the modulation and maintenance of epidermal homeostasis by regulating genes critical for adhesion, differentiation and proliferation of stratified epithelial cells (Chikh et al., 2011). The role of ASPP family proteins in the nervous system has also been reported (Sottocornola et al., 2010). ASPP2 interacts with Par3 and plays a key role in controlling neural progenitor cell proliferation, polarity and brain structure during central nervous system development. iASPP is highly expressed in monkey embryonic stem cells and downregulated during neuronal differentiation (Wang et al., 2018), but its role in neuronal development remains inexplicit.

miR-124 is a miRNA specifically expressed in the nervous system, accounting for more than 20% of the total miRNA in the brain (Lagos-Quintana et al., 2002) and promoting neuronal differentiation (Makeyev et al., 2007; Gu et al., 2014). miR-124 is not expressed or has a low level of expression in neural stem cells, but is abundantly expressed in the postmitotic neurons (Cheng et al., 2009). Many studies have confirmed that iASPP is a direct target gene of miR-124 in the cancer context and under neuropathological conditions (Liu et al., 2013; Chen et al., 2014; Liang et al., 2017; Liu et al., 2018), suggesting that this interaction may have important roles in early neuronal development.

Here, we investigated the role of iASPP in neuronal development and showed that the expression of iASPP is downregulated during neuronal differentiation. Silencing the expression of iASPP promoted neurite development, while overexpression of iASPP inhibited neuronal cell development. We also explored the molecular mechanisms underlying iASPP regulation of neuronal development and found that iASPP may associate with some important proteins involved in neuronal development and inhibit the phosphorylation of the cytoskeleton protein β-II spectrin (Sptbn1) by directly binding to the α-II spectrin (Sptan1). Overexpression of the Phosphorylation site mutant form of sptbn1 influence neurite development.

## Materials and methods

### Reagents and antibodies

All-trans-retinoic acid (atRA) and Ara-C were purchased from Sigma Chemical Co. (Sigma, Munich, Germany). Dithio-bis succinimidylpropionate (DSP) was purchased from Thermo Fisher Scientific (Waltham, MA, United States). The following antibodies were used: iASPP (Sigma, 1:2000), Flag (Proteintech Group, Chicago, United States 1:2000), Myc (Proteintech, 1:2000), His (Proteintech, 1:2500), pan-phospho-serine antibody (Santa Cruz Biotechnology, Santa Cruz, CA, 1:2000), GFP (Cell Signaling Technology Inc., Beverly, MA, United States, 1:2000) and Tubulin (Sigma, 1:5000).

### Cell culture and transfection

The HEK293T, N2A and P19 cell lines were purchased from the American Type Culture Collection (ATCC). HEK293T and N2A cells were maintained in Dulbecco’s modified Eagle’s medium (DMEM; Thermo) supplemented with 10% fetal bovine serum (FBS; Thermo). N2A cells were transfected in 24-well plates using TurboFect transfection reagent (Thermo) according to the manufacturer’s protocol. N2A cell neuronal differentiation was induced by culturing N2A cells in DMEM containing 1% FBS and 10 μM atRA for 48 h or 72 h. P19 cells were maintained in α-modified minimum essential medium (α-MEM; Thermo) supplemented with 10% FBS. atRA-induced P19 cell differentiation was performed according to our previous procedures (Gu et al., 2018). Briefly, P19 cells were cultured in suspension to form embryoid bodies (EBs) in bacterial-grade Petri dishes at a seeding density of 1 × 10^5^ cells/ml in the presence of 1 μM RA with fresh medium changed every other day. After 4 days of aggregation, the embryoid bodies were dissociated into single cells by trypsin–EDTA (Thermo) digestion and plated in poly-L-lysine-coated tissue culture dishes at a density of 2 × 10^3^ cells/cm^2^ in Neurobasal-A medium (Thermo) with 2% B27 supplement (Thermo) for neuronal differentiation. Primary cortical neurons were prepared from embryonic day 16–17 ICR mouse pups as described (Hilgenberg and Smith, 2007). The cells were cultured in poly-L-lysine-coated 24-well plates at a density of 150,000 cells/well in Neurobasal medium (Thermo) supplemented with 2% B27 (Thermo), 0.5 mM glutamine, penicillin and streptomycin. One day post plating, the neurons were transfected with Lipofectamine 2,000 reagent (Thermo) according to the manufacturer’s instructions. Cell morphology was examined 48 h or 72 h post-transfection, and neurite length was measured using ImageJ software (NeuronJ). Neurites were manually selected and traced semiautomatically to measure the neurite length. A neuron was scored as having neurites if it harbors processes that are longer than two cell body diameters.

### Plasmid construction

The cDNAs of mouse iASPP and iASPP, PP1ca, Sptan1 and Sptbn1 with various tags were cloned into the pLVX-IRES-tdTomato plasmid (Clontech, Takara Bio, Japan) plasmid with tag sequences in the reverse primers. The iASPP interference and iASPP Overexpression plasmid for puromycin selection of the stably infected cells were cloned using pLVshRNA-EGFP(2A)-Puro (Inovogen, Beijing, China) and pCDH-CMV-MCS-EF1-CopGFP-Puro (System Biosciences, Mountain View, CA), respectively. A validated GFP-MAP1B plasmid (Scales et al., 2009) was a gift from Phillip Gordon-Weeks (Addgene plasmid # 44396). The Sptbn1-mutated and Map1b-mutated expression vectors were generated using the QuikChange Site-Directed Mutagenesis Kit (Stratagene, La Jolla, CA). The iASPP-Cherry construct consisting of Cherry fused to the C-terminal of iASPP was generated using pmCherry-N1 (Clontech). All the related primers are shown in Supplementary Table S1.

### Lentivirus production

Recombinant lentiviral particles were produced in HEK293T cells. At 70–80% confluence, cells were transfected with independent lentiviral plasmids, together with the helper plasmids pCMV-VSV-G, pRSV-REV, and pMDL using TurboFect transfection reagent, following the manufacturer’s instructions. The virus-containing medium was harvested 72 h post-transfection and filtered using 0.45-μm filter. Lentiviral transduction was performed by incubating P19 cells with 1 ml virus-containing medium with 5 μg/ml polybrene in a 24-well culture plate overnight. Cells were then replaced with fresh culture medium for 24 h followed by selection in culture medium containing 1 μg/ml puromycin for 5–7 days with the fresh medium changed every other day.

### Immunocytochemistry

P19 cells that were subjected to neuronal differentiation for 48 h or 72 h were fixed with 4% paraformaldehyde (PFA) for 20 min, permeabilized and blocked in phosphate buffer solution (PBS) with 0.3% TritonX-100 and 5% bovine serum albumin (BSA), and then incubated with the Tuj1 antibody at 4°C overnight. The cells were stained for 1 h with Alexa 595-conjugated secondary antibody (Thermo, 1:1000).

### Immunohistochemistry

Immunohistochemistry was carried out on paraffin-embedded 5-μm-thick coronal sections of embryonic mouse brains. Brain sections were washed and blocked in PBS supplemented with 5% BSA and 0.1% Triton X-100 for 30 min at room temperature followed by incubation with primary antibodies against iASPP (Sigma, 1:500), Pax6 (Proteintech, 1:500) or Tuj1 (Cell Signaling Technology, 1:500) overnight at 4°C. After washing, sections were incubated with the Alexa-conjugated secondary antibody (Thermo, 1:1000) for 1 h at 37°C and then counterstained with DAPI (Sigma, 1:1000) for 20 min at room temperature.

### *In situ* hybridization

*In situ* hybridization of the brain sections was performed with digoxigenin-labeled antisense riboprobes. cDNA of iASPP was amplified with specific PCR primers (Supplementary Table S1) and cloned into the PSPT18 vector (Roche) to generate an antisense probe for iASPP. The digoxigenin-labeled antisense riboprobes were synthesized by *in vitro* transcription using SP6 Riboprobe System (Roche). Mouse brains were fixed in 4% paraformaldehyde (PFA) for 4–6 h. The brain tissues were then embedded in paraffin, and paraffin-embedded brains were cut into 8–10 μm sections. Brain sections were hybridized for 18 h at 42°C. The hybridization signal was detected with an alkaline phosphatase-coupled antibody (1,1,000) against digoxigenin, as well as nitroblue tetrazolium and 5-bromo-4-chloro-3-indolyl phosphate as color reaction substrates.

### CoIP

For the CoIP experiment, cell lysates from N2A cells were homogenized in RIPA lysis Buffer (Beyotime Institute of Biotechnology, Shanghai, China), immunoprecipitated with antibodies overnight at 4°C and incubated with Protein G-Agarose (20 μL, Santa Cruz Biotechnology) for 2 h at 4°C. Immunoprecipitates were collected, and the Sepharose was resuspended in RIPA Buffer, washed five times and incubated in SDS sample buffer for 10 min at 95°C.

### LC–MS/MS analysis

Whole cells were treated with 2 mM the membrane-permeable cross-linker DSP for 30 min at room temperature, lysed in RIPA lysis buffer and immunoprecipitated with the indicated anti-tag antibodies overnight at 4°C, followed by incubation with protein G beads for 2 h at room temperature. All protein sample analysis for LC–MS/MS were performed by Biotech Pack Scientific (Beijing, China). Identified proteins that interacted with iASPP were available in Supplementary Table S2 and proteins that were dephosphorylated by iASPP-PP1 complex are listed in Supplementary Table S3.

### Immunoblotting

Cells were lysed using RIPA buffer containing protease inhibitors (Beyotime). The protein concentrations of the extracts were measured with a BCA assay kit (Beyotime). Equal amounts of denatured samples were loaded and subjected to SDS–PAGE, transferred onto polyvinylidene difluoride (PVDF) membranes (Millipore, Bedford, MA), probed with secondary antibodies against corresponding primary antibodies and detected using immobilon western chemiluminescent HRP substrate (Millipore Corporation, Billerica, MA).

### Statistical analysis

All experiments were repeated at least two times. Neurite lengths were determined using ImageJ software (NIH). A single neurite was manually selected and traced semiautomatically to measure neurite length. Identified neuronal cells were scored as neurite-harboring cells if a process greater than two cell body diameters in length was observed. The results were analyzed using one-way ANOVA combined with multiple comparisons for more than two groups comparison or unpaired t-tests for two groups comparison to determine the significant differences between the experimental groups, and a *p* value <0.05 was considered statistically significant.

## Results

### The expression of iASPP was downregulated during neuronal development

**Figure 1:**
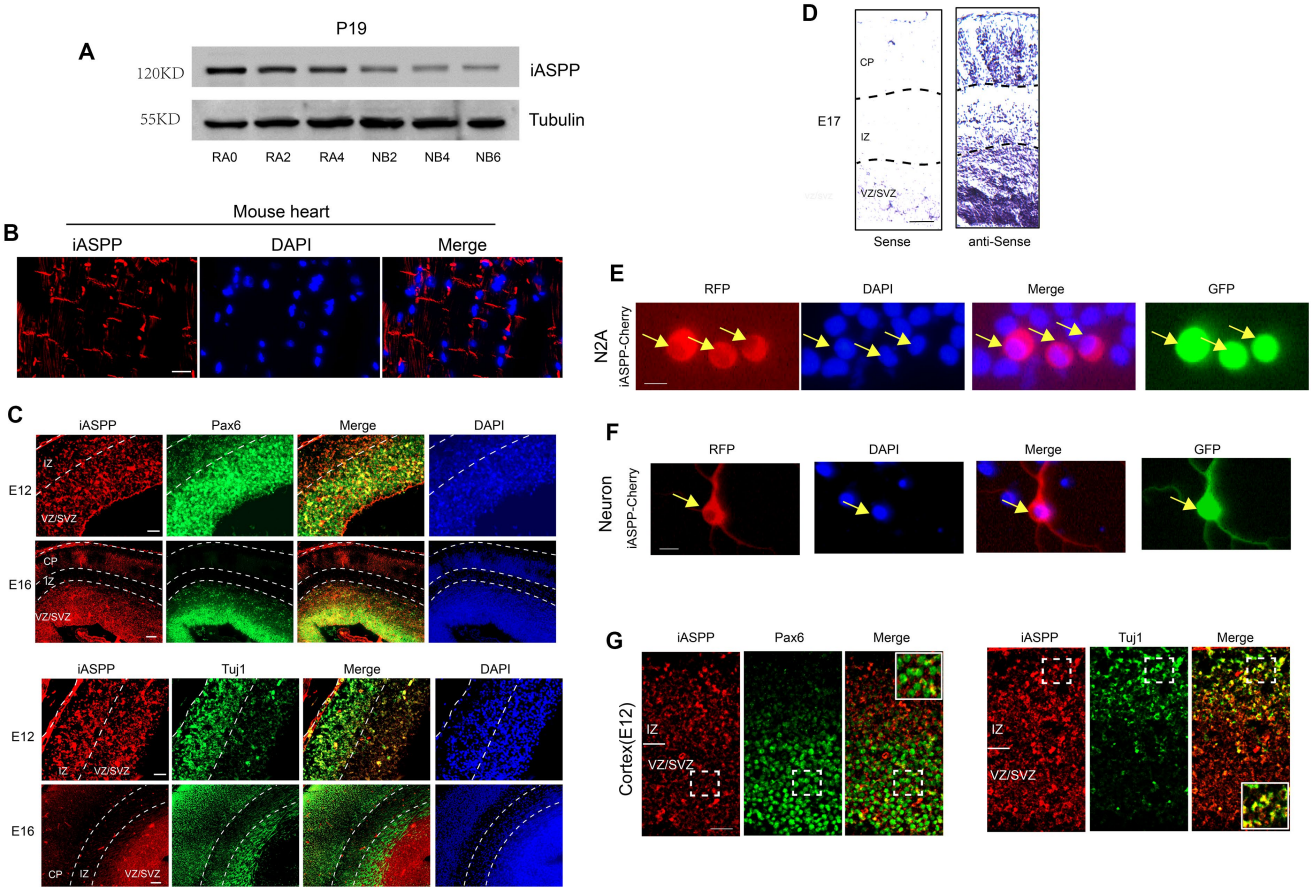
iASPP expression is downregulated during neuronal development. **(A)** Immunoblotting reveals that iASPP expression is decreased during P19 cell neuronal differentiation. RA0, RA2 and RA4 represent the 0th, 2nd and 4th day of neuronal induction of P19 cells under the stimulation of retinoic acid and NB2, NB4 and NB6 represent the 2nd, 4th and 6th day of neuronal differentiation of P19 cells in the neuronal differentiation medium following retinoic acid induction. **(B)** Immunofluorescence showed that iASPP was expressed in the intercalated disc structure in mouse heart, indicating the effectiveness of the antibody **(C)** Immunofluorescence staining shows that iASPP is highly expressed in the VZ and the SVZ of developing mouse cortex. **(D)** *In situ* hybridization of embryonic mouse brain sections with an iASPP-specific probe indicates that iASPP mRNA is decreased during brain development. **(E)** RFP monitoring of iASPP location in N2A cells. **(F)** RFP monitoring of iASPP location in cortical neurons. **(G)** iASPP is expressed in the cytoplasm of E12 mouse cortex. Scale bars, 100 **(D)**, 50 **(B,C,G)**, 20 **(E,F)** μm.

We first examined the expression of iASPP in the neuronal differentiation model of teratoma P19 cells. P19 cells have a variety of common properties with embryonic stem cells and can differentiate into neurons after retinoic acid (RA) induction (Mummery et al., 1990). We examined the changes in iASPP expression during this process. P19 cells were induced by RA for 4 days and differentiated in differentiation medium for another 6 days. Samples were collected every other day. The results showed that the expression of iASPP was stronger in P19 cells before induction, and gradually decreased during neuronal induction and differentiation (Figure 1A).

We then investigated the expression patterns of iASPP in the developing embryonic mice brain. A previous study indicated that iASPP is highly expressed in the heart and is mainly located in the intercalated disc structure of human and mouse myocardial fibers (Notari et al., 2015). Based on these expression characteristics of iASPP, we first performed immunohistochemistry in the heart tissue of ICR mice with the iASPP antibody we used. Consistent with previous findings, iASPP was expressed on the intercalated disc structure located at the longitudinal ends of cardiomyocytes with no obvious nonspecific signals (Figure 1B), implying that iASPP antibody specifically identified iASPP in mouse tissues with our experimental procedure.

We next analyzed the expression pattern of iASPP in the embryonic day 12 (E12) and E16 mouse cortex. The results showed that in the early stage of neurogenesis (E12), when the cortical plate (CP), which is formed by migrating neurons generated from neural stem cells, had not yet clearly formed and the neural stem cells existed in the whole cortex, the iASPP signal was present in the entire cortex. With the development of cortex, the CP is formed in the later stage of neurogenesis (E16), and iASPP was mainly expressed in the Pax6-positive neural progenitor cell region, the ventricular zone (VZ) and subventricular zone (SVZ) and downregulated in the Tuj1-positive neurogenic region (Figure 1C). These results suggested that iASPP may play a role in neural stem cell maintenance and that its downregulation during cortical development may promote neuronal differentiation.

We further studied the expression pattern of iASPP mRNA in the cerebral cortex of E17 mouse brains by *in situ* hybridization. The results showed that iASPP mRNA was highly expressed in the VZ and SVZ and dramatically decreased in the IZ and CP (Figure 1D), which indicated that the expression of iASPP mRNA gradually decreased during the process of neuronal differentiation of neural stem cells.

To investigate the localization of iASPP in neuronal cells, we generated an overexpression plasmid, named iASPP-Cherry, by fusing the coding region of iASPP with the red fluorescent protein Cherry. N2A cells and primary cortical neurons were co-transfected with iASPP-Cherry and a GFP-expressing plasmid and observed by fluorescence microscopy 48 h later. The results showed that iASPP was expressed in the cytoplasm of N2A cells and primary neurons (Figures 1E,F). In addition, we investigated the subcellular localization of iASPP in the E12 fetal mouse cerebral cortex *in vivo* by immunohistochemistry. The results also showed cytoplasmic localization, as demonstrated by the co-localization of iASPP with Tuj1, a neuronal marker expressed in the cytoplasm, but not with Pax6, a neural stem cell marker expressed in the nucleus (Figure 1G) that was consistent with its expression in the adult brain (Liu et al., 2013).

### IASPP inhibited neuronal development

**Figure 2:**
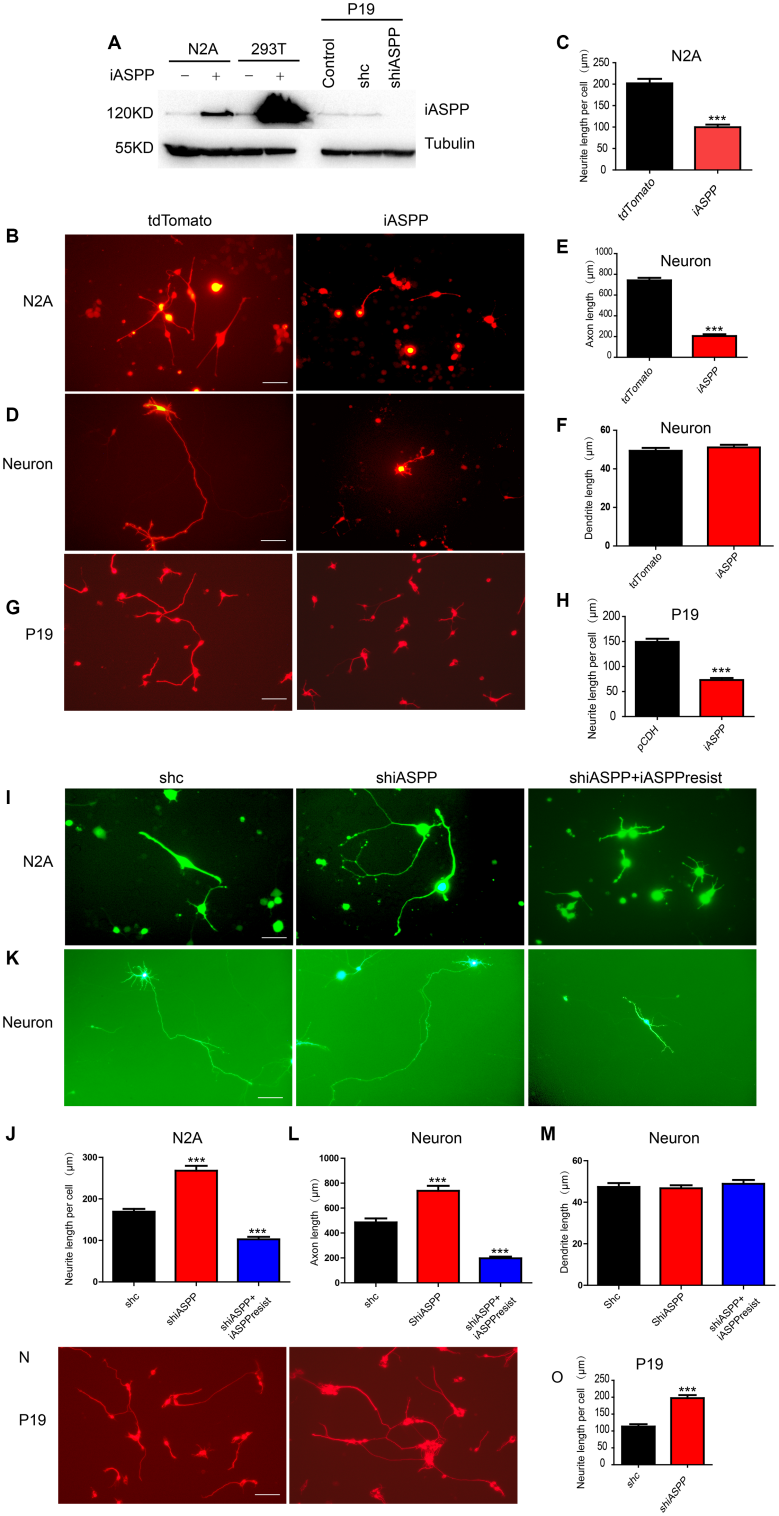
iASPP inhibits neuronal differentiation. **(A)** Immunoblotting for iASPP expression shows that Tomato-iASPP and shiASPP increase and reduce the amount of iASPP, respectively. **(B–H)** iASPP overexpression suppresses neuronal differentiation in various cellular models. The plasmids indicated in the figure are able to express the nonfusion form of full-length iASPP and the corresponding fluorescent protein separately. **(I–O)** iASPP knockdown promotes neuronal differentiation in various cellular models. The morphologies of N2A cells and cortical neurons are observed by monitoring the fluorescent protein expression in the successfully transfected cells, and the morphology of P19 cells is observed by immunofluorescence staining of the neuronal marker Tuj1 using fluorescence microscopy. Scale bars, 50 μm. Mean values (*n* = 10 to 20 cells) ± s.e.m. (^***^p < 0.001).

To explore the role of iASPP in neuronal differentiation, we generate plasmids to overexpress or knockdown the expression of iASPP and named as iASPP and shiASPP, respectively. The effectiveness of these constructs was confirmed by immunoblotting with transfection of 293 T and N2a cells for iASPP and puromycin-selected P19 cells, primary cortical neurons and N2A cells for shiASPP (Figure 2A, Supplementary Figures S1A-F). We transfected the iASPP plasmid and control plasmid into N2A cells and mouse primary cortical neurons, respectively. The IRES element in the plasmid ensures the expression of fluorescent protein separately from the inserted genes. After further 72 h of neuronal differentiation, the cell morphology was observed by monitoring fluorescent protein by fluorescence microscopy. The results showed that iASPP overexpression significantly inhibited neurite elongation in N2A cells and axon but not dendrite growth in primary neurons (Figures 2B–F). Furthermore, we performed neuronal differentiation experiments with iASPP stably expressing P19 cells and determined the neurite length 72 h after neuronal differentiation through immunocytochemistry (Tuj1 staining). The results showed that the overexpression of iASPP significantly inhibited the neurite growth of differentiated P19 cells (Figures 2G,H). These data suggested that the iASPP inhibited neurite development during neuronal differentiation.

To further demonstrate the role of iASPP in neuronal development, we knocked down the endogenous iASPP expression in N2A cells, cortical neurons and P19 cells with the shiASPP plasmid. The results showed that knocking-down the expression of iASPP promoted the neurite elongation in N2A and P19 cells and stimulated axon but not dendrite growth in primary neurons 48 h after further neuronal differentiation, which could be blocked by co-transfection of plasmid expressing a shRNA-resistant iASPP (Figures 2I–O). The above results suggested that iASPP had an inhibitory effect on neuronal development and that the decreased expression of iASPP during neuronal differentiation promoted neuronal development.

### iASPP interacted with Sptan1

**Figure 3:**
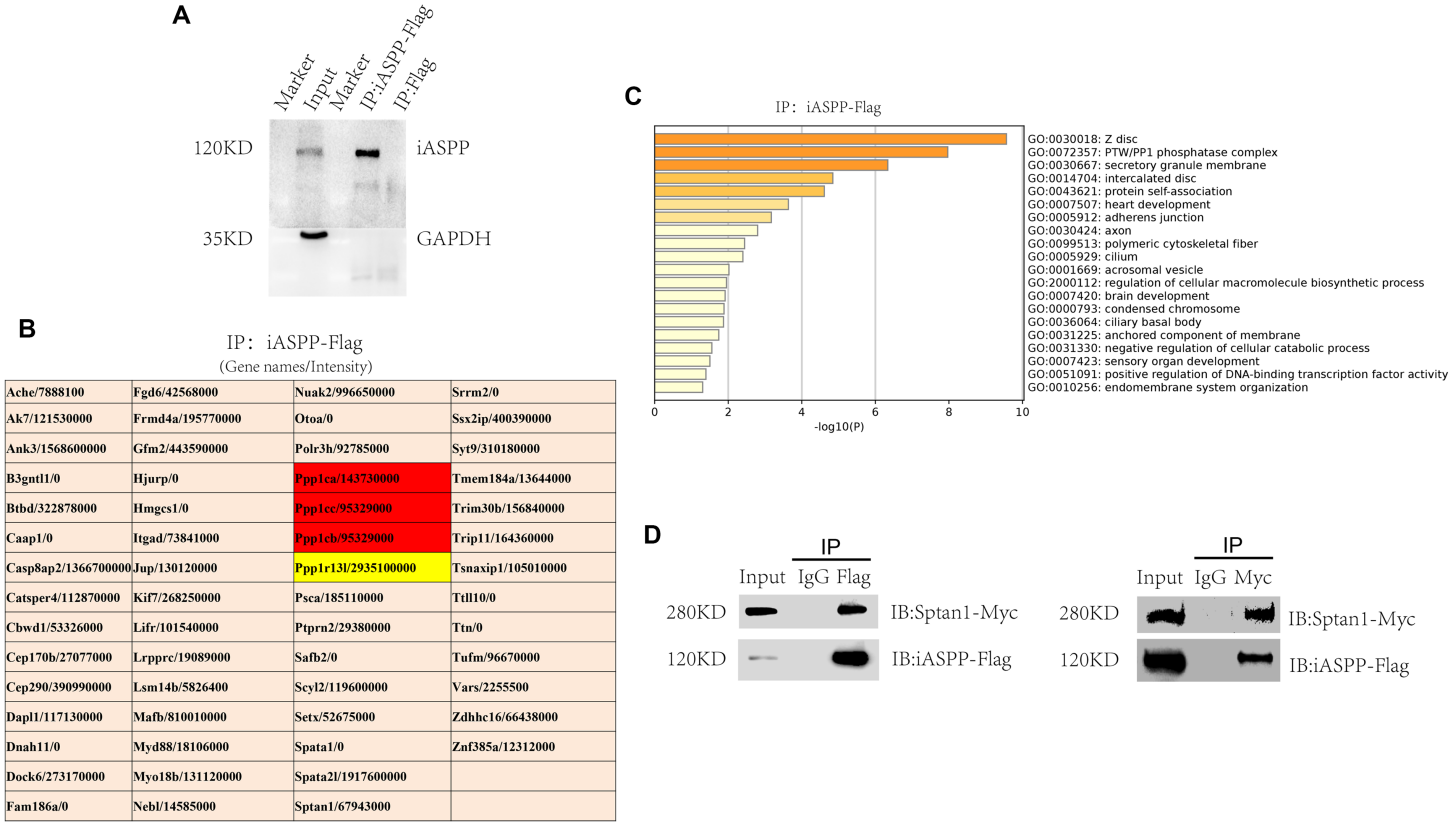
Sptan1 is a partner of iASPP. **(A)** Confirmation of iASPP-Flag pulldown by IP with an anti-Flag antibody. **(B)** A list of proteins identified by CoIP-MS that bind with iASPP, excluding the overlapping proteins identified by iASPP-Flag and the control group as well as IgG-related components. **(C)** Graph showing pathway enrichment analysis of iASPP binding proteins. **(D)** Reciprocal coimmunoprecipitations were carried out to further confirm the interaction of iASPP with Sptan1.

To explore the molecular mechanisms underlying the regulation of neuronal development by iASPP, we next systematically investigated proteins that interacted with iASPP. We constructed an iASPP overexpression plasmid with the coding region of iASPP fused with a Flag tag in the C-terminal to generate iASPP-Flag. N2A cells were transfected with iASPP-Flag and the control plasmid tdTomato-Flag, and neuronal differentiation was induced 48 h later. After another 48 h, Flag-tagged iASPP and its binding proteins were pulled-down by CoIP and the successful enrichment of iASPP-Flag was confirmed by immunoblotting (Figure 3A).

Next, the eluate was analyzed by using liquid chromatography-mass spectrometry (LC–MS). As expected, the most abundant protein detected was iASPP. In addition, the different catalytic subunits of phosphatase PP1, namely, Ppp1ca, Ppp1cb and Ppp1cc, which have been confirmed to be able to interact with iASPP in previous studies, were detected in the iASPP-Flag pulldown group but not the control pulldown group (Figure 3B; Supplementary Table S2) (Llanos et al., 2011; Bertran et al., 2019). Then, pathway and process enrichment analysis of iASPP interaction proteins was performed using the Metascape bioinformatics online tool^1^ and the most enriched biological pathways and processes were associated with the PP1 phosphatase complex, Z disc, intercalated disc, heart development and adhesion junction (Figure 3C), which was consistent with the report that iASPP was expressed in cardiomyocytes, colocalized with junctional proteins in the intercalated disc and participated in the regulation of myocardial function (Notari et al., 2015). In addition, iASPP immunoprecipitated proteins may also participate in the regulation of axon and brain development (Figure 3C), indicating that iASPP may interact with some neuronal-related proteins to regulate nervous system development.

Sptan1 is a structural protein that is widely distributed in the brain. When Sptan1 is genetically deleted, the axon initial segment structure is damaged, and both dendrites and axons are abnormal (Wang et al., 2018). In addition, Sptan1 plays an important role in maintaining the normal morphology of neuron cell bodies and stimulating neurite growth (Trinh-Trang-Tan et al., 2014). The abnormal expression of Sptan1 may cause early infantile epileptic encephalopathy, intellectual disability, speech impediment and autism (Hernandez et al., 2022). Since Sptan1 has also been detected by mass spectrometry in studies of the iASPP interactome by other researchers (Mangon et al., 2021), we decided to further investigate the interaction between iASPP and Sptan1 and its function in neuronal development. We made a Sptan1 expression plasmid with the C-terminus of the Sptan1 coding region fused with a Myc tag to generate Sptan1-Myc. N2A cells were cotransfected with iASPP-Flag and Sptan1-Myc, and the cells were lysed 48 h later for CoIP analysis. The results showed that ectopically expressed iASPP and Sptan1 coprecipitated with each other. Neither iASPP-Flag nor Sptan1-Myc was detected in the negative control (IgG) group, which further confirms the MS result (Figure 3D).

### Sptan1 recruited Ppp1ca through iASPP and regulated Sptbn1 and Map1b phosphorylation

**Figure 4:**
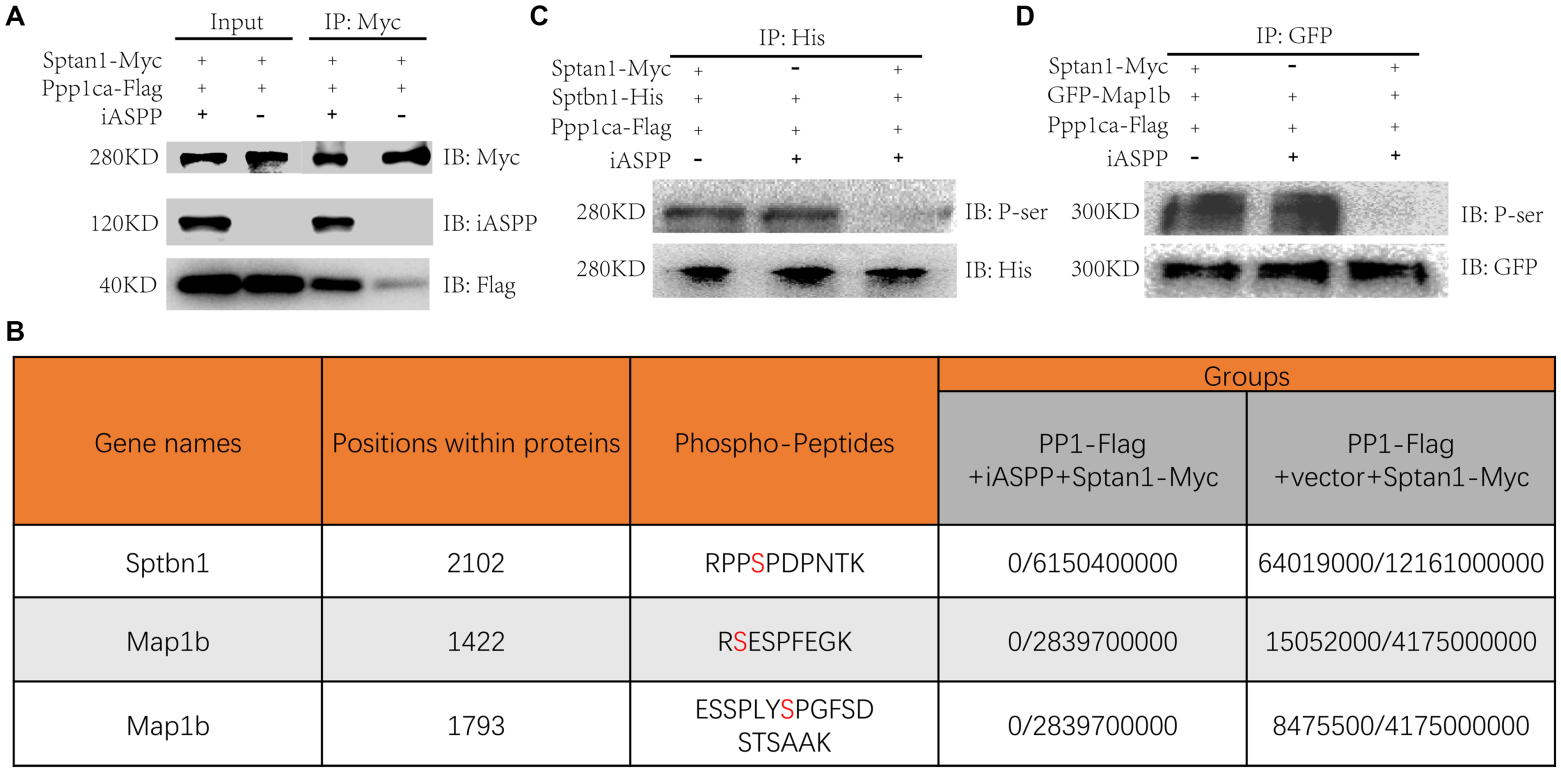
iASPP dephosphorylates Sptbn1 by recruiting the iASPP-PP1 complex. **(A)** Coimmunoprecipitations were carried out to investigate the interaction of Sptan1 with the iASPP-PP1 complex. **(B)** Table depicting the detection of phosphorylated peptides of the corresponding genes in each group (Phospho-peptide intensity/total peptide intensity). The phosphorylation sites in peptides are marked in red color. **(C)** Immunoblotting for phospho-Sptbn1 expression shows that iASPP modulates Sptbn1 phosphorylation. The indicated plasmids were transfected into N2A cells. Protein lysates were divided into three parts: one for the Input and the other for the indicated antibody IP. The immunoprecipitated were probed with anti-His antibodies to detect Sptbn1-His and a pan-phospho-serine antibody to detect phosphorylated Sptbn1-His. **(D)** Immunoblotting for phospho-Map1b expression shows that iASPP modulates Sptbn1 phosphorylation. The indicated plasmids were transfected into N2A cells. Protein lysates were divided into three parts: one for the Input and the other for the indicated antibody IP. The immunoprecipitated were probed with anti-GFP antibodies to detect GFP-Map1b and a pan-phospho-serine antibody to detect phosphorylated GFP-Map1b.

Serine/threonine protein phosphatase-1 (PP1) is an important intracellular protein phosphatase that regulates protein function by dephosphorylation, thereby influencing cell division, muscle contraction, gene expression, glycogen metabolism, neural signal transduction, and other physiological processes (Aggen et al., 2000). The formal name of the gene encoding the iASPP protein is “protein phosphatase 1 regulatory subunit 13 like” (PPP1R13L), suggesting a potential function as a PP1 regulatory subunit. Indeed, direct interactions between iASPP and PP1 have been confirmed by separate studies (Bergamaschi et al., 2003; Llanos et al., 2011), and we detected the three catalytic subunits of PP1 in our CoIP/MS experiment (Figure 3B; Supplementary Table S2). However, the role of the iASPP-PP1 complex in neurodevelopment is not clear; therefore, we hypothesized that the iASPP-PP1 complex may regulate Sptan1 function by dephosphorylating the serine/threonine residues of Sptan1. Thus, we constructed a PP1 catalytic subunit α expression plasmid with a Flag tag fused at the end of the coding region, named Ppp1ca-Flag. Ppp1ca-Flag and Sptan1-Myc were cotransfected with iASPP or the control plasmid tdTomato into N2A cells, and the effects of the iASPP-PP1 complex on the phosphorylation of Sptan1 were studied by CoIP experiments followed by mass spectrometry. The results indicated that by pulling-down Sptan1-Myc in the iASPP coexpression group, significantly more Ppp1ca-Flag was detected in the eluant in addition to iASPP compared with the control group (Figure 4A), suggesting that sptan1 might recruit PP1 by binding to iASPP.

To investigate whether the binding of the iASPP-PP1 complex to Sptan1 modulates the phosphorylation of Sptan1, we performed a mass spectrometry-based phosphorylation site analysis on the eluents from the CoIP experiment described above. While we did not find any phosphorylated serine/threonine site on Sptan1 in the Sptan1-Myc group, additional proteins were detected by mass spectrometry, including Sptbn1, a binding partner of Sptan1, and Map1b, a microtubule-associated protein, which plays an important role in nervous system development. Unexpectedly, the phosphorylation levels of several serine residues in both proteins were downregulated (Figure 4B; Supplementary Table S3). These results suggested that by recruiting the iASPP-PP1 complex, Sptan1 may regulate the phosphorylation and function of both proteins. Next, we further analyzed whether Sptbn1, which interacts with Sptan1 (Machnicka et al., 2012), is dephosphorylated by iASPP. We constructed an overexpression plasmid of Sptbn1 with a His tag (Sptbn1-His) and then expressed it with Sptan1-myc, Ppp1ca-Flag and iASPP or the control vector in N2A cells, respectively. Forty-eight hours later, His tagged-Sptbn1 was pulled-down by IP, and then the phosphorylation state of Sptbn1 was detected by a pan-phospho-serine antibody. The results showed a significant reduction in the phosphorylation of Sptbn1 in the iASPP-expressing group compared to the control group that lacking iASPP or Sptan1 overexpression (Figure 4C). The same results were obtained in the GFP-Map1b transfection experiment (Figure 4D). Furthermore, through CoIP experiment, we did not find a direct interaction between iASPP and Sptbn1 (Supplementary Figure S2A). The above results indicated that the iASPP-PP1 complex may dephosphorylate Sptbn1 and Map1b by binding to Sptan1.

**Figure 5:**
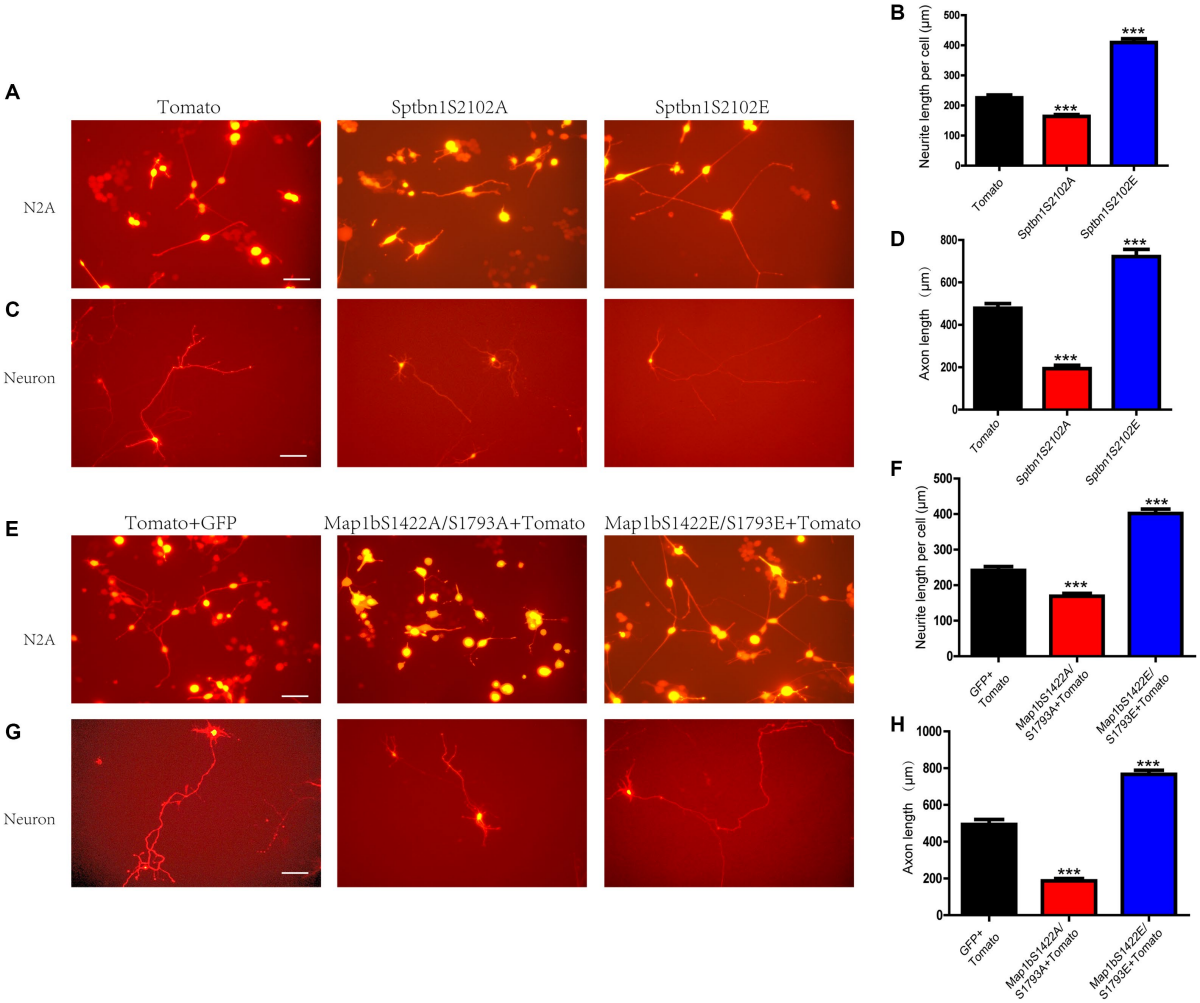
Sptbn1 (2102S) and Map1b (S1422, S1793) phosphorylation is essential for neuronal differentiation. **(A**–**D)** non-phosphorylated form of Sptbn1 (Sptbn1-S2102A) inhibits while phosphomimetic form of Sptbn1 (Sptbn1-S2102E) promotes both N2A cell and cortical neuron differentiation. **(E–H)** non-phosphorylated form of Map1b (Map1b-S1422A/S1793A) inhibits while phosphomimetic form of Map1b (Map1b-S1422E/S1793E) promotes both N2A cell and cortical neuron differentiation. The morphologies of the differentiated N2A cells and cortical neurons were observed by monitoring the red fluorescent protein expression of the successfully transfected tdTomato-positive cells using fluorescence microscopy. Scale bars, 50 μm. Mean values (*n* = 10 to 20 cells) ± s.e.m. (^***^p < 0.001).

We next investigated the role of these phosphorylation regulation in neuronal development. The Ser2102 residue of Sptbn1-His and Ser1422/1793 of GFP-Map1b residues were replaced with alanine or glutamate to create non-phosphorylated or phosphomimetic forms, respectively, and transfected them into N2A cells or 48 h intro culturing primary cortical neurons and the neuronal morphology was observed under a fluorescence microscope 48 h later. The results showed that the overexpression of the non-phosphorylated form of Sptbn1 or Map1b inhibited while overexpression of the phosphomimetic form of Sptbn1 or Map1b stimulated neurite development of N2A cells and axon but not dendrite growth of cortical neurons (Figures 5A–H; Supplementary Figure S3A,B), suggesting that iASPP-mediated dephosphorylation of these proteins may at least partially contributed to the inhibitory effect of iASPP on neurite development.

## Discussion

**Figure 6:**
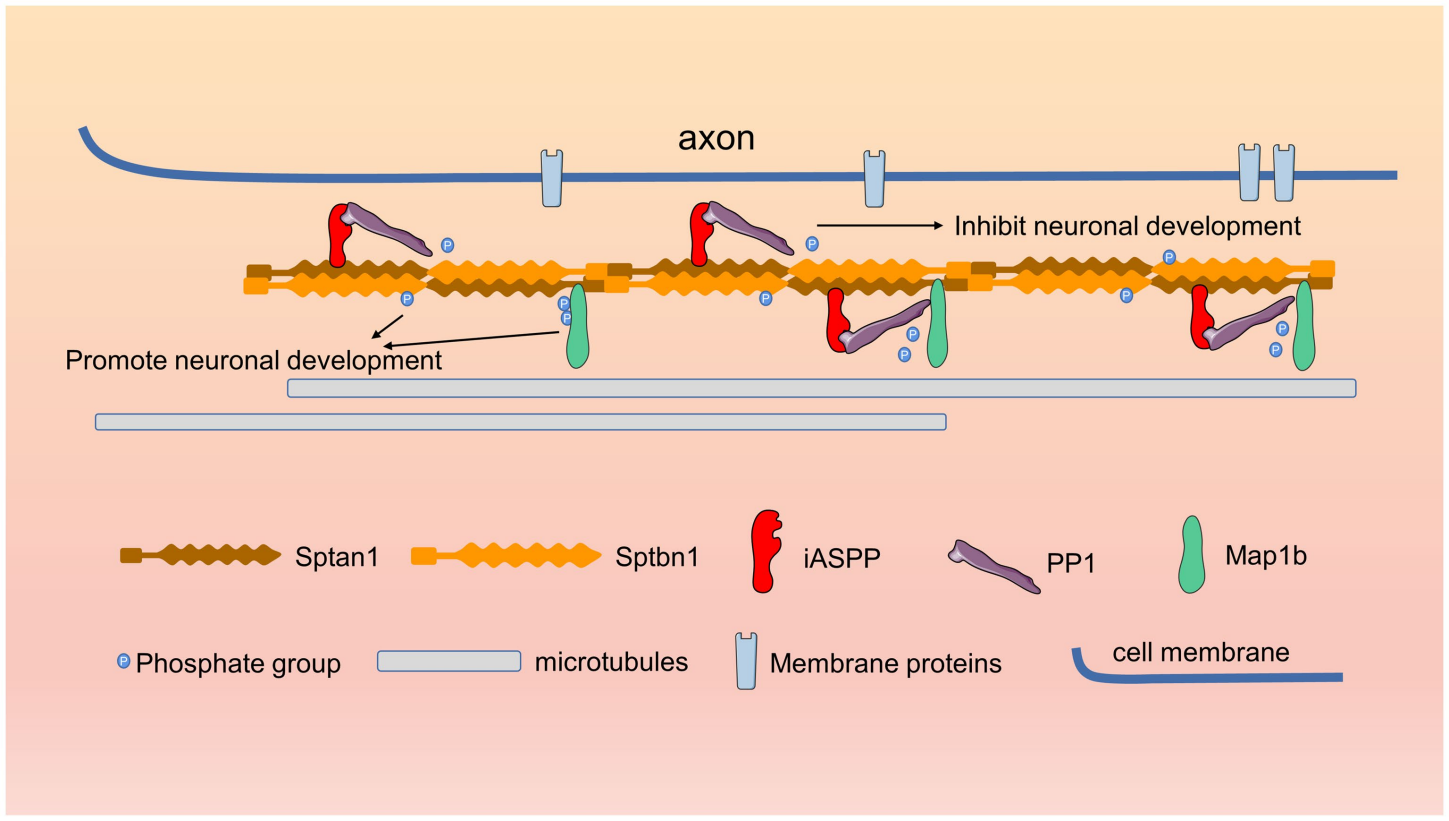
Schematic of iASPP-regulated neuronal differentiation. During neuronal development, iASPP binds directly to the cytoskeletal protein Sptain1 and recruits PP1, which dephosphorylates Sptbn1 and Map1b, which directly bind to Sptan1, thereby inhibiting neurite development.

This study demonstrated that the expression of iASPP decreased progressively during neuronal differentiation and that iASPP inhibited neuronal cell neurite development by regulating the phosphorylation state of the cytoskeletal component spectrin protein (Figure 6).

miR-124 is a brain-specific miRNA whose role in neuronal development and neuronal cell function has been intensively studied. The expression of miR-124 increases during the differentiation of neural stem cells to neurons and miR-124 promotes neuronal differentiation by inhibiting the expression of many neurodevelopmental inhibitors (Makeyev et al., 2007; Visvanathan et al., 2007; Cheng et al., 2009; Neo et al., 2014). A substantial body of literature, most of which involves tumor or neuropathological conditions, demonstrates that iASPP is a direct target gene of miR-124 (Liu et al., 2013; Chen et al., 2014; Liang et al., 2017; Liu et al., 2018). Our previous study showed that miR-124 inhibited the expression of iASPP in the human neuronal cell line M17 cells and that iASPP overexpression inhibited the miR-124-mediated neuronal differentiation of M17 cells (Lin et al., 2014). In this study, we further investigated the expression and role of iASPP in neuronal development. Our research showed that the expression of iASPP protein was gradually decreased during P19 cell neuronal differentiation, which is contrary to the expression pattern of miR-124 previously confirmed by us and other researchers in this cellular model (Neo et al., 2014; Gu et al., 2018) and consistent with the conclusion that miR-124 inhibits the expression of iASPP. The expression of the iASPP protein and mRNA gradually decreased from VZ/SVZ to CP in embryonic mice brain, indicating that the expression of the iASPP gene gradually decreases during neuronal differentiation *in vivo*. Previous studies have demonstrated that miR-124 expression is low in the VZ/SVZ and high in CP (Makeyev et al., 2007) and this complementary expression of miR-124 and iASPP in the developing brain further supports the conclusion that iASPP is a direct target of miR-124.

We investigated the role of iASPP in neuronal development by overexpression and knockdown of iASPP in a mouse N2A cell neuronal differentiation model, a P19 cell neuronal differentiation model, and mouse embryonic primary cortical neurons. The results showed that overexpression of iASPP significantly inhibited the neurite development, while knockdown of endogenous iASPP expression significantly promoted neurite outgrowth, indicating that iASPP inhibited the neurite growth during neuronal differentiation. These conclusions are also consistent with our previous study on neuronal differentiation in human M17 cells (Lin et al., 2014). A study has shown that ASPP2 promotes the establishment of neuroepithelial cell polarity and the migration and differentiation of neurons by interacting with Par3 (Sottocornola et al., 2010), which is largely contrary to the role of iASPP suggested by our study, indicating that the opposing role between ASPP and iASPP may exist in various cellular contexts. In addition, studies have shown that genetic variation of the chromosomal region in which iASPP is located may lead to neurodevelopmental diseases (Castillo et al., 2014; Rim et al., 2017), suggesting that iASPP might be studied as a candidate gene for these diseases in further research.

Our CoIP/MS analysis suggests that iASPP may directly associate with some proteins that are involved in nervous system development and neurodevelopmental disorders, such as Ank3 and Sptan1. As a cell membrane-cytoskeleton adapter protein, Ank3 may participate in axon initial segment organization, Ranvier junction ion channels and cell adhesion molecule structure maintenance (Leussis et al., 2012). The genetic variation of Ank3 is closely related to neurodevelopmental diseases such as autism spectrum disorder and intellectual disability (Bi et al., 2012; Iqbal et al., 2013). Sptan1 is also an important cytoskeletal protein that participates in the construction of the axon initial segment and the abnormal expression of Sptan1 may lead to early infantile epileptic encephalopathy, intellectual disability, aphasia and autism (Huang et al., 2017). Indeed, previous studies have reported that iASPP is involved in the regulation of the cytoskeleton. Mangon et al. found that iASPP interacts with an important microtubule regulatory protein, the microtubule positive end-binding protein EB1, to regulate the positioning of the spindle and chromosome segregation in mitotic cells (Mangon et al., 2021). In addition, our MS results failed to detect P53 and RelA/p65, which were previously demonstrated to bind to iASPP (Bergamaschi et al., 2003; Ge et al., 2021), which was consistent with the localization of iASPP in the cytoplasm in our neuronal cellular context and further indicated that iASPP could act on diverse signaling pathways in various physiological processes.

As a ubiquitously expressed phosphatase, PP1 regulates the function of target proteins through dephosphorylation. Previous studies have established that iASPP may act as a regulatory subunit of PP1, participating in the interactions between PP1 and other proteins (Gao et al., 2018; Zhou et al., 2019). We found that Sptan1 regulates the phosphorylation of the Sptbn1 protein by recruiting the iASPP-PP1 complex. Sptbn1 can form a tetramer with Sptan1 at distal axons and regulate axonal growth, neuronal polarity and protein transport and maintain synaptic structural stability by interacting with the cytoskeleton component actin (Sihag et al., 1996; Galiano et al., 2012; Wang et al., 2018; Kalichamy et al., 2020; Cousin et al., 2021; Yang et al., 2021). Sptbn1 is composed of two Calponin homology (CH) domain, 17 spectrin repeats (SRs) and one pleckstrin homology (PH) domain from the N-terminus to the C-terminus. The CH domain is responsible for the interaction with the cytoskeleton component microfilaments (Yang et al., 2021). The SR structure is found in many cytoskeletal proteins, including spectrin, alpha-actin, dystrophin, etc., and is involved in the regulation of the organization, stability and morphology of the cell membrane, and is integral in connecting the cell membrane system with the main cytoskeleton dynamic transport system (Djinovic-Carugo et al., 2002). The PH domain is responsible for connecting Sptbn1 to the membrane system of cell organelles (Das et al., 2008). We found that the phosphorylated site of Sptbn1 (S2102) that was affected by iASPP was in the last SR. Previous studies found that mutations in the Sptbn1 SRs may lead to developmental delay, autism and intellectual disability (Rosenfeld et al., 2021), which means that the SRs are crucial to the function of Sptbn1. We found that the elimination of phosphorylation modification in the last SR of Sptbn1 hinders neuronal process development, further suggesting the importance of the Sptbn1 SR structure in the regulation of Sptbn1 function. Of course, although our study did not find a regulatory effect of iASPP on the phosphorylation of Sptan1, we cannot rule out the possibility that iASPP may directly regulate Sptan1 function. It is necessary to study the interactions closer to the physiological state of embryonic development. In addition, the coverage of protein amino acid residues detected by mass spectrometry may also affect the identification of phosphorylation changes in a protein.

Through mass spectrometry analysis and CoIP experiment, we also revealed that overexpression of iASPP reduced the phosphorylation state of microtubule-associated protein Map1b. Studies have shown that Map1b is highly expressed in the brain during embryonic development, and its high phosphorylation state is important for early nervous system development (Black et al., 1994). Studies have also shown that many proteins, including Map1b, contain proline (P)-directed serine/threonine phosphorylation sites in the axonal growth cones (Pigino et al., 1997). The phosphorylation sites of Map1b regulated by iASPP that we found all fall into this category, further suggesting that iASPP may play important roles in regulating neuronal development. .

Taken together, we report that iASPP knockdown promotes neuronal development by modulating the phosphorylation state of various cytoskeletal related proteins and these findings will strengthen our understanding of the molecular mechanism of cytoskeletal regulation in the developing brain.

## Data availability statement

The datasets presented in this study can be found in online repositories. The names of the repository/repositories and accession number(s) can be found in the article/Supplementary material.

## Ethics statement

The animal work was performed in strict accordance with the recommendations described in the Guide for the Care and Use of Laboratory Animals of the National Institute of Health. The protocol was approved by the Committee on the Ethics of Animal Experiments of Fujian Medical University (Permit Number: FJMU IACUC 2018-089).

## Author contributions

XG and CJ designed all the experiments and wrote the manuscript. JW, QG and JZ finished all the experiments. All authors contributed to the article and approved the submitted version.

## Funding

This work was supported by the Natural Science Foundation of China (82271198), the Joint Funds for the Innovation of Science and Technology of Fujian province (2018Y9068), the Natural Science Foundation of Guangdong province (2018A030313008), the Natural Science Foundation of Fujian Province (2020 J01598), and the funds of Fujian Medical University (XRCZX 2019009).

## Conflict of interest

The authors declare that the research was conducted in the absence of any commercial or financial relationships that could be construed as a potential conflict of interest.

## Publisher’s note

All claims expressed in this article are solely those of the authors and do not necessarily represent those of their affiliated organizations, or those of the publisher, the editors and the reviewers. Any product that may be evaluated in this article, or claim that may be made by its manufacturer, is not guaranteed or endorsed by the publisher.

## Footnotes

^1^ www.metascape.org

## Supplementary material

Supplementary Figure S1 Knockdown efficiency of shiASPP on iASPP protein expression in different cells. Immunoblotting for iASPP expression showed that shiASPP reduced the amount of iASPP in puromycin selected P19 cells (**A**, **B**), primary cortical neurons (**C**, **D**) and N2A cells (**E**, **F**). (n = 3 independent experiments; bar represents mean values ± s.e.m. (**P* < 0.05, ****P* < 0.001; α-Tubulin served as loading control).

Supplementary Figure S2 iASPP does not interact with Sptbn1 directly. (**A**) Sptbn1-His was not immunoprecipitated by iASPP-Flag and vice versa.

Supplementary Figure S3 Sptbn1 (2102S) and Map1b (S1422, S1793) phosphorylation is not essential for dendrite differentiation of primary cortical neurons. The bar graph shows the length of the dendrite in vitro of normal and phosphorylate-sites mutant form of Sptbn1 (**A**) and Map1b (**B**) transfected cells.
