# Supplementary figures and images for: iASPP regulates neurite development by interacting with Spectrin proteins
Source: Front Mol Neurosci. 2023 May 22;16:1154770. doi: 10.3389/fnmol.2023.1154770 (PMC10240065; doi:10.3389/fnmol.2023.1154770)

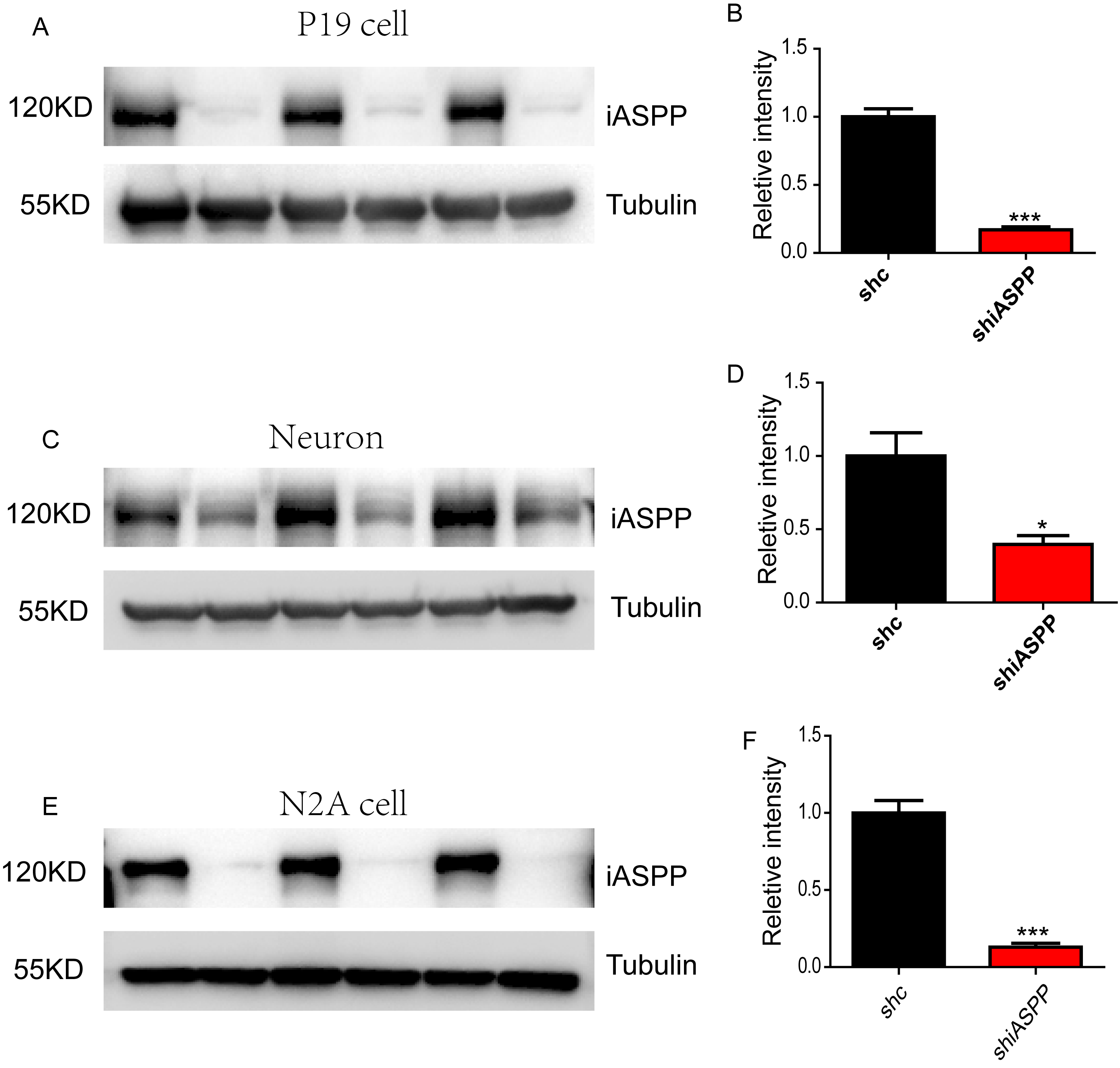

Supplement: Supplementary Figure S1 — Knockdown efficiency of shiASPP on iASPP protein expression in different cells. Immunoblotting for iASPP expression showed that shiASPP reduced the amount of iASPP in puromycin selected P19 cells (A, B), primary cortical neurons (C, D) and N2A cells (E, F). (n = 3 independent experiments; bar represents mean values ± s.e.m. (*P < 0.05, ***P < 0.001; α-Tubulin served as loading control). [file Image_1.tif]

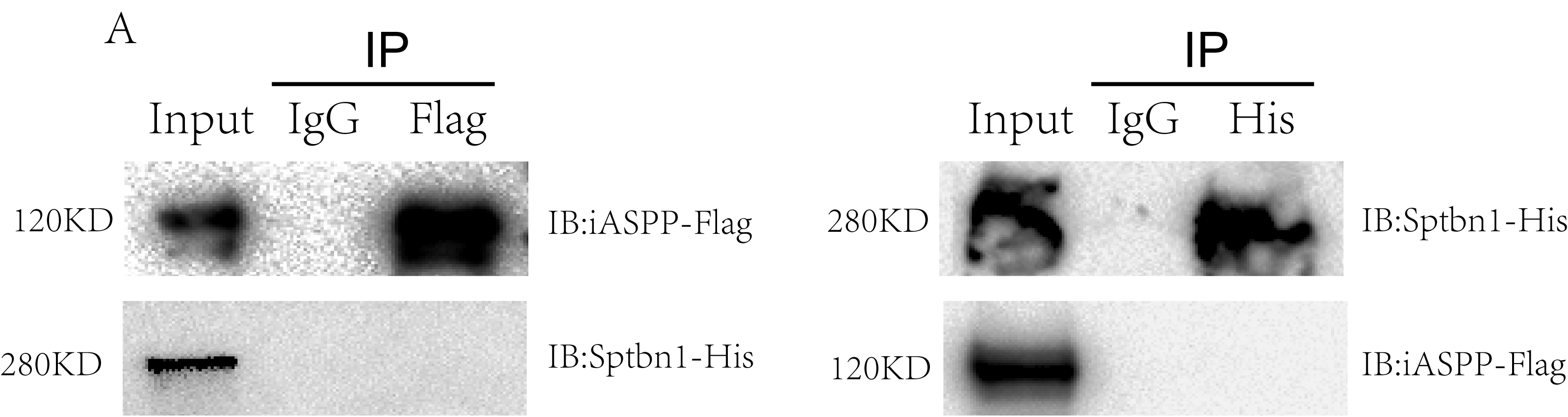

Supplement: Supplementary Figure S2 — iASPP does not interact with Sptbn1 directly. (A) Sptbn1-His was not immunoprecipitated by iASPP-Flag and vice versa. [file Image_2.tif]

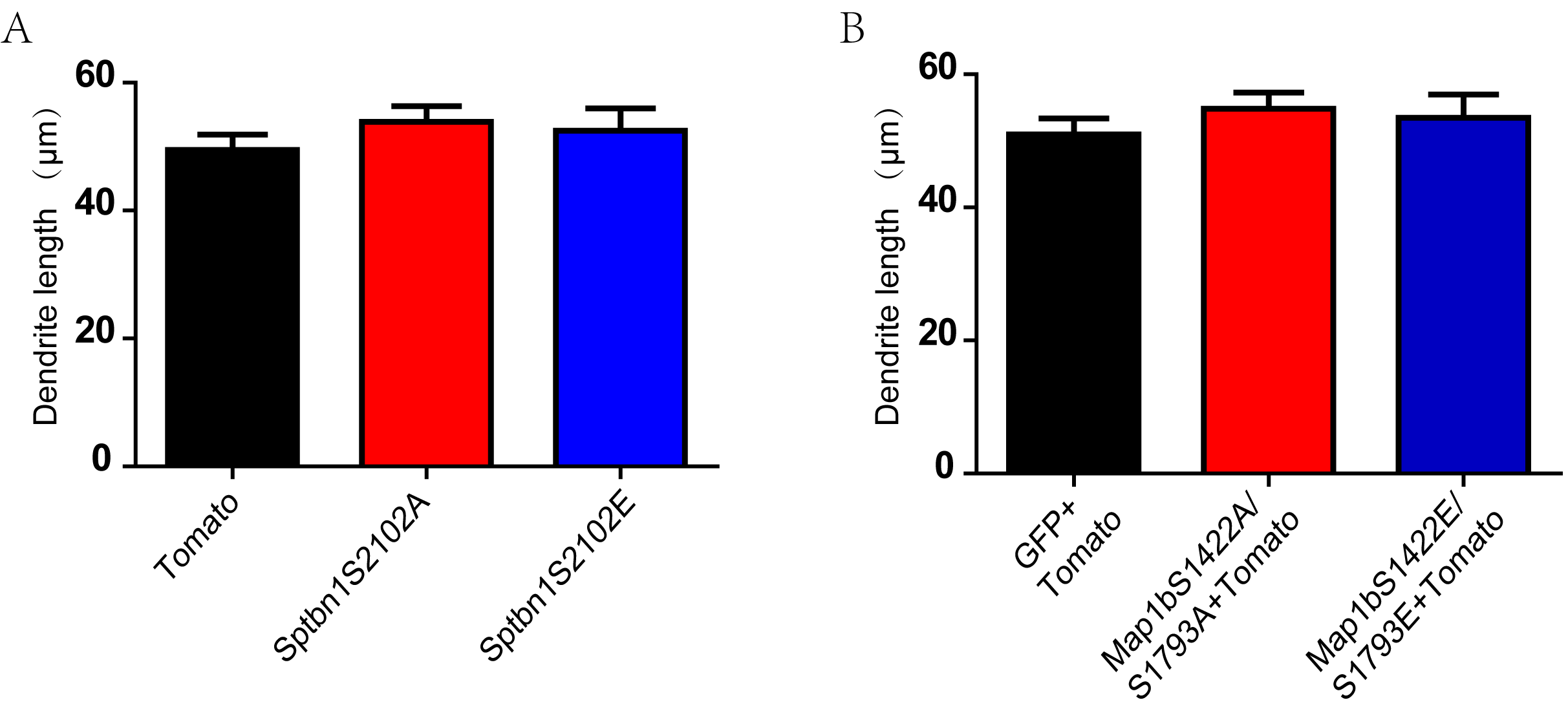

Supplement: Supplementary Figure S3 — Sptbn1 (2102S) and Map1b (S1422, S1793) phosphorylation is not essential for dendrite differentiation of primary cortical neurons. The bar graph shows the length of the dendrite in vitro of normal and phosphorylate-sites mutant form of Sptbn1 (A) and Map1b (B) transfected cells. [file Image_3.tif]
