# Supplementary material for: iASPP regulates neurite development by interacting with Spectrin proteins
Source: Front Mol Neurosci. 2023 May 22;16:1154770. doi: 10.3389/fnmol.2023.1154770 (PMC10240065; doi:10.3389/fnmol.2023.1154770)
Supplement: Supplementary file 7 [file Table_4.docx]

GraphPad Prism 5.01-Statistics and generation of statistical charts（contain data tables）

https://www.jianguoyun.com/p/DarFDJ4QtK-uCxiokvQEIAA

Microscopy images

https://www.jianguoyun.com/p/DTpgWyEQtK-uCxiukvQEIAA

Western blot images

<https://www.jianguoyun.com/p/DX07owgQtK-uCxi1kvQEIAA>

The protein interactions data have been submitted to the IMEx (http://www.imexconsortium.org) consortium through IntAct [X] and assigned the identifier - IM-29688

https://www.ebi.ac.uk/intact/editor/service/export/mi/publication-imex?imex=IM-29688&format=html&conversationContext=1
